# Huntingtin CAG repeats in neuropathologically confirmed tauopathies: Novel insights

**DOI:** 10.1111/bpa.13250

**Published:** 2024-02-28

**Authors:** Sergio Pérez‐Oliveira, Juan Castilla‐Silgado, Cèlia Painous, Iban Aldecoa, Manuel Menéndez‐González, Marta Blázquez‐Estrada, Daniela Corte, Cristina Tomás‐Zapico, Yaroslau Compta, Esteban Muñoz, Albert Lladó, Mircea Balasa, Gemma Aragonès, Pablo García‐González, Maitée Rosende‐Roca, Mercè Boada, Agustín Ruíz, Pau Pastor, Beatriz De la Casa‐Fages, Alberto Rabano, Raquel Sánchez‐Valle, Laura Molina‐Porcel, Victoria Álvarez

**Affiliations:** ^1^ Laboratory of Genetics Hospital Universitario Central de Asturias Oviedo Spain; ^2^ Instituto de Investigación Sanitaria del Principado de Asturias (ISPA) Oviedo Spain; ^3^ Department of Functional Biology (Physiology) University of Oviedo Oviedo Spain; ^4^ Parkinson's Disease and Movement Disorders Unit, Department of Neurology Hospital Clinic of Barcelona Barcelona Spain; ^5^ UB Neuro Institut de Neurociències, Maeztu Center University of Barcelona Barcelona Spain; ^6^ Fundació de Recerca Clínic Barcelona‐Institut d'Investigacions Biomèdiques August Pi i Sunyer (FRCB‐IDIBAPS) Barcelona Spain; ^7^ Neurological Tissue Bank of the Biobank‐Hospital Clinic‐FRCB‐IDIBAPS Barcelona Spain; ^8^ Pathology Department, Biomedical Diagnostic Center Hospital Clínic de Barcelona, University of Barcelona Barcelona Spain; ^9^ Department of Neurology Hospital Universitario Central de Asturias Oviedo Spain; ^10^ Department of Medicine University of Oviedo Oviedo Spain; ^11^ Biobank of Principado de Asturias, Hospital Universitario Central de Asturias (HUCA) Oviedo Spain; ^12^ Alzheimer's Disease and other Cognitive Disorders Unit Neurology Service, Hospital Clínic, FRCB‐IDIBAPS, University of Barcelona Barcelona Spain; ^13^ Ace Alzheimer Center Barcelona – Universitat Internacional de Catalunya Barcelona Spain; ^14^ Networking Research Center on Neurodegenerative Diseases (CIBERNED) Instituto de Salud Carlos III Madrid Spain; ^15^ Unit of Neurodegenerative Diseases, Department of Neurology University Hospital Germans Trias i Pujol and The Germans Trias i Pujol Research Institute (IGTP) Badalona Barcelona Spain; ^16^ Movement Disorders Unit, Department of Neurology Hospital General Universitario Gregorio Marañón Madrid Spain; ^17^ Instituto Investigación Sanitaria Gregorio Marañón Madrid Spain; ^18^ Neuropathology Department and Brain Tissue Bank CIEN Foundation, Queen Sofia Foundation Alzheimer Center Madrid Spain

**Keywords:** Alzheimer disease, corticobasal degeneration, *HTT* gene, progressive supranuclear palsy, tauopathies

## Abstract

Previous studies have suggested a relationship between the number of CAG triplet repeats in the *HTT* gene and neurodegenerative diseases not related to Huntington's disease (HD). This study seeks to investigate whether the number of CAG repeats of *HTT* is associated with the risk of developing certain tauopathies and its influence as a modulator of the clinical and neuropathological phenotype. Additionally, it aims to evaluate the potential of polyglutamine staining as a neuropathological screening. We genotyped the *HTT* gene CAG repeat number and APOE‐ℰ isoforms in a cohort of patients with neuropathological diagnoses of tauopathies (*n*=588), including 34 corticobasal degeneration (CBD), 98 progressive supranuclear palsy (PSP) and 456 Alzheimer's disease (AD). Furthermore, we genotyped a control group of 1070 patients, of whom 44 were neuropathologic controls. We identified significant differences in the number of patients with pathological *HTT* expansions in the CBD group (2.7%) and PSP group (3.2%) compared to control subjects (0.2%). A significant increase in the size of the *HTT* CAG repeats was found in the AD compared to the control group, influenced by the presence of the Apoliprotein E (APOE)‐ℰ4 isoform. Post‐mortem assessments uncovered tauopathy pathology with positive polyglutamine aggregates, with a slight predominance in the neostriatum for PSP and CBD cases and somewhat greater limbic involvement in the AD case. Our results indicated a link between *HTT* CAG repeat expansion with other non‐HD pathology, suggesting they could share common neurodegenerative pathways. These findings support that genetic or histological screening for *HTT* repeat expansions should be considered in tauopathies.

## Graphical abstract

Assessment of neuropathological findings in individuals affected by pathological expansion of CAG *HTT*.

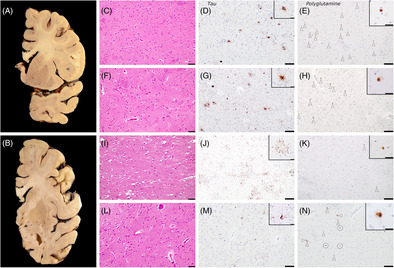

## 1 INTRODUCTION

Tauopathies are a heterogeneous group of neurodegenerative disorders characterized by abnormal microtubule‐associated protein (tau) deposition within the cerebral tissue. Tau binds to tubulin and plays a role in promoting the assembly and stabilization of microtubules. The six isoforms of tau are distinguished by the presence or absence of 29‐ or 58‐amino‐acid inserts in the N‐terminus domain and the presence of either three (3R tau isoforms) or four (4R tau isoforms) tandem repeat sequences of 31 or 32 amino acids [1].

Abnormal accumulation of tau is a pathological hallmark of many neurodegenerative diseases, such as progressive supranuclear palsy (PSP), corticobasal degeneration (CBD), and Alzheimer's disease (AD). The 4R tau isoform predominates in PSP and CBD, whereas AD combines 3R and 4R isoforms. The brain exam from patients with tau protein aggregations often shows concurrence of many other neurodegenerative diseases with amyloid‐beta, alpha‐synuclein, and TDP‐43 pathology [2, 3]. Moreover, increased huntingtin (HTT) immunoreactivity in AD patients' neuronal cells of post‐mortem brains have also been shown [4].

Huntington's disease (HD) is an inherited neurodegenerative disease linked to a CAG repeat expansion at the *Huntingtin* gene *(HTT*; MIM:613004). CAG *HTT* allele expansions above 35 can cause HD, and above 39 are fully penetrant. CAG *HTT* intermediate alleles (IAs; range 27‐35) are unstable and are prone to increase their length into a pathological range in the offspring. Interestingly, HD has also been proposed to be a tauopathy [5]. First, the mutant HTT protein alters tau splicing, phosphorylation, oligomerization and subcellular localization [6, 7]; second, patients with HD present aggregated tau inclusions within various structures of the brain, including those with young‐onset [8, 9] and, third, the MAPT H2 haplotype influences the cognitive function of HD patients [9, 10].

Recently, a link between the CAG repeats in the *HTT* gene and frontotemporal dementia/amyotrophic lateral sclero (FTD/ALS) phenotypes has been proposed [11]. Additionally, we have reported that IAs in *HTT* can have a role in AD and FTD risk [12, 13] and synucleinopathies [14].

Unraveling the genetic architecture of tauopathies is crucial for designing clinical trials and developing new drugs and disease‐modifying interventions. Therefore, the main objective of this study is to determine whether the HTT CAG repeats in the HTT gene are associated with the risk of developing tauopathies. Additionally, we aim to perform a neuropathological analysis of *HTT* inclusions in the brains of pathological expansion carriers. To accomplish this, we analyze a case‐control series that only includes patients with neuropathologically confirmed tauopathies.

## 2 METHODS

### 2.1 Subjects

This is a multicenter study that includes brain samples of patients (*n* = 588) obtained from the Neurological Tissue Bank (NTB) of the Hospital Clinic‐FRCB‐IDIBAPS (Barcelona, Spain) and from the Principado de Asturias BioBank (Oviedo, Spain, PT17/0015/0023). Also, samples and data from some patients included in this study were provided by the Biobank Banco de Tejidos Fundación CIEN. These samples were processed following standard operating procedures with the appropriate approval of the Ethics and Scientific Committees. The Neuropathological diagnosis of AD, PSP and CBD was performed according to standard international consensus criteria [15, 16, 17]. Early‐onset AD (EOAD) was defined as AD with clinical manifestations starting before 60 years to ensure the selection of early‐onset cases, given the study's retrospective nature [18]. Late‐onset Alzheimer Disease (LOAD) refers to cases where clinical manifestations begin at or after 65 years of age [19].

Furthermore, we included a control cohort consisting of 1070 subjects. Among them, 44 individuals originated from the NTB‐FRCB‐IDIBAPS and had undergone neuropathological studies. The remaining participants comprised clinical controls (*n* = 1026) recruited from two outpatient clinics: Hospital Universitario Central de Asturias (Oviedo, Spain) and Ace Alzheimer Center (Barcelona, Spain). Gender, age at onset, the age of the last visit, and the age of death were collected. All the participants or legal representatives gave written informed consent to participate in the study, which the Ethical Committees of Hospital Universitario Central de Asturias and collaborating centers approved.

### 2.2 Genetic analysis

Genomic DNA was isolated from the brain tissue of patients (cerebellum) or peripheral blood leukocytes (control population) following standard procedures. In patients carrying IAs or pathological expansions in the *HTT* gene, we also obtained DNA from the putamen, caudate, and frontal cortex to study somatic mosaicism.

*HTT* gene CAG repeat number was analyzed by a polymerase chain reaction with 50‐fluorescence labeled primers [20]. The number of repeats was determined by capillary electrophoresis using an ABI 3130X automated DNA sequencer and the *GeneMapper* version 4.0 software (Applied Biosystems, Foster City, CA, USA). To provide size standards, several samples with different *HTT* CAG allele lengths were sequenced as positive controls. In individuals with *HTT* expanded alleles, Sanger sequencing was performed to analyze the loss of CAA interruption, which is associated with early onset HD and it is particularly relevant to individuals carrying alleles in the reduced penetrance range (i.e., 36–39 CAG repeats) [21]. In terms of interpretation of *HTT* CAG repeats, the shorter allele was called “allele 1” and the longer allele “allele 2” (Figure S1). APOE‐Ɛ isoforms (SNPs rs7412 + rs4293589) were genotyped with a real‐time PCR *Taqman*® custom assay (Applied Biosystems, Foster City, CA, USA) as described [22].

### 2.3 Neuropathological study

The neuropathological examination was performed according to standardized protocols at the NTB. Half the brain hemisphere was dissected in coronal sections and frozen, while the other half was fixed by immersion in 4% formalin for 3 weeks. At least 25 representative brain areas were embedded in paraffin, cut at 5 μm, and stained with hematoxylin & eosin (H&E). Immunohistochemistry (IHC) was performed using various antibodies including anti‐βA4 (6F/3D, Dako, Glostrup, Denmark), anti‐tau (AT8, Thermo Scientific, USA), anti‐α‐synuclein (KM51, Leica, Germany; 5G4, Analytik Jena, Germany), anti‐ubiquitin (P4D1, Cell Signaling, USA), anti‐α‐internexin (2E3, Invitrogen, USA), anti‐TDP‐43 (2E2‐D3, Abnova, Taiwan), and anti phospho TDP‐43 (11‐9, Cosmo Bio Co, Japan). All IHC were counterstained with hematoxylin.

A retrospective evaluation of available clinical data and stains was conducted in cases of tauopathies with pathological expansions in the *HTT* gene. The neuropathological diagnosis was updated based on current criteria. Assessment of atrophy, neuronal loss, and gliosis was performed in the striatum/head of the caudate nucleus to determine if they exceeded the expected levels observed in the neuropathological processes secondary to their tauopathy. In these subjects, we also studied the presence and pattern of distribution of polyglutamine deposits. To do this, 5 μm formalin‐fixed, paraffin‐embedded sections from the frontal cortex, striatum globus pallidus, hippocampus, midbrain, and cerebellum were stained with polyglutamine antibody (5TF1‐1C2, 1/7000, Millipore, MA, USA). A Leica Bond Max platform was used, with 20 minutes of pretreatment with Leica ER1 (citrate based) and 60 minutes of primary antibody incubation. Four subjects diagnosed with HD (>39 *HTT* expansions) were added to the analysis, as well as six subjects diagnosed with PSP (*n* = 2), CBD (*n* = 2), and AD (*n* = 2) but without pathological *HTT* expansions (<21 *HTT* expansions). The neuropathological assessment was performed by LMP and IA blinded to the diagnosis and the number of *HTT* CAG repeats.

### 2.4 Statistical analyses

To compare the frequency of the IAs and pathological *HTT* among groups, Chi‐square and Fisher's tests were performed. To analyze the influence of expanded IAs on the disease onset, the Kruskal–Wallis test was performed followed by, with their corresponding pairwise comparisons Holm adjustment, after verifying by means of the *Kolmogorov–Smirnov* test that the variable did not follow a normal distribution. To determine the relationship between the *HTT* CAG IAs and the survival probability a Kaplan–Meier method and the log‐rank test were used. Survival was calculated as the time from symptom onset to death from any cause (outcome = 1) or censoring date (outcome = 0). Categorical variables were described in frequencies and percentages. Mean and standard deviation were used for the analysis of continuous variables. To assess the effect of predictors on specific diseases, multivariate logistic regression models were created, including all the independent factors available. For the matrix‐type correlation analysis among quantitative variables (age onset, age at death, and survival), Spearman's rank correlation test was used.

Multiple comparison corrections were made by pairwise comparisons for categorical variables (normal, intermediate or expanded *HTT* CAG allele, death rate and gender) and Dunn's Multiple Comparison Test for quantitative variables (disease group, *HTT* CAG repeat number, age at onset, age at last visit/death and disease duration). Cox regression models, multivariate and linear regression models were run to find the predictors of age at onset and death, as well as survival or development risk of the neuropathologies. Statistical analysis was performed using the R statistical software (version 4.0.5) and SPSS Statistics (version 19).

## 3 RESULTS

### 3.1 Subjects

**FIGURE 1:**
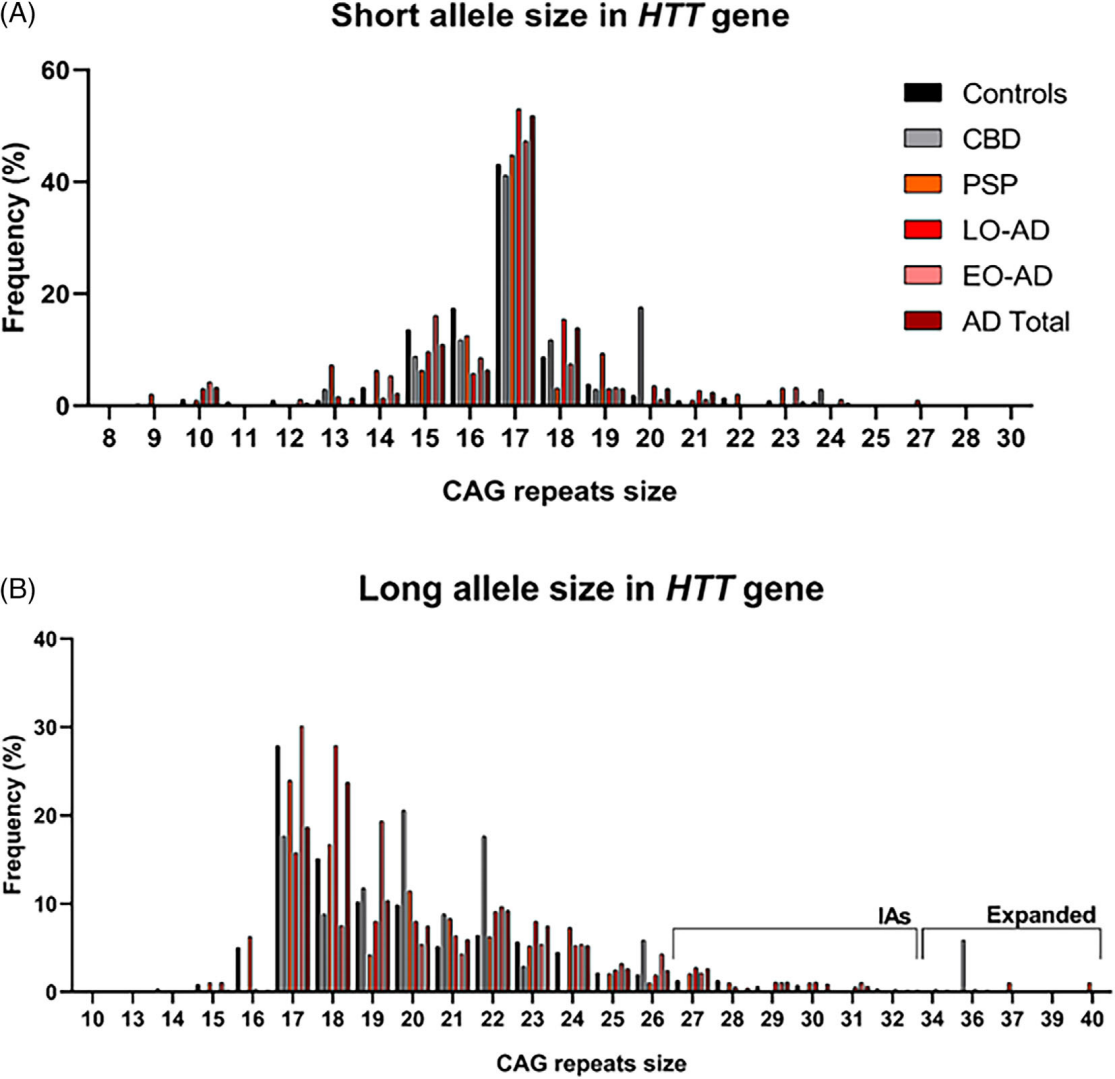
Distribution of HTT CAG alleles across the sample. AD, Alzheimer disease; CBD, corticobasal degeneration; Controls, healthy controls; EO, early‐onset Alzheimer disease; LO‐AD, late‐onset Alzheimer disease; PSP, progressive supranuclear palsy. *Statistically significant *p* < 0.05 (all comparisons are made between the neuropathology and the control cohort). *N*= total genotyped.

*HTT* gene CAG repeats were analyzed in 1658 subjects. Among the 588 neuropathological cases with disease, 34 were diagnosed with CBD, 98 with PSP, and 456 with AD (363 with LOAD and 93 with EOAD). The control group comprised 1070 unrelated healthy subjects, of which 44 were neuropathological samples. Controls did not have any neurological condition (Figure 1).

There was a female predominance among the LOAD group (68.9%) compared with control (53.6%; *p* < 0.001; OR[95% CI]:1.91[1.47–2.49]), PSP (41.2%; *p* < 0.001; OR[95% CI]:3.14[1.94–5.14]) and EOAD (43.5%; *p* < 0.001; OR[95% CI]:0.35[0.21–0.57]) groups, whereas the gender was equally balanced among the other groups.

Age at onset was statistically different between AD, CBD, and PSP groups (*p* < 0.005), with LOAD patients experiencing the latest disease onset (74.28 ± 7.05 years). EOAD showed a longer disease duration (10.79 ± 4.86 years) compared with the LOAD (9.36 ± 4.96 years; *p* < 0.05) and PSP (8.21 ± 3.91 years; *p* < 0.001). Age at death was statistically different between all groups (*p* < 0.05) by having the LOAD patients the latest age at death (83.40 ± 7.04 years) (Table 1).

**TABLE 1:** Demographic data of the cohorts studied.

A							
	CBD (*n* = 34)	PSP (*n* = 98)	AD (*n* = 456)	LOAD (*n* = 363)	EOAD (*n* = 93)	Controls (*n* = 1070)	Sig.
Gender (Female)	21 (61.8%)	40 (41.2%)	290 (63.7%)	250 (68.9%)*	40 (43.5%)	560 (53.6%)	*p* < 0.05; LO‐AD versus Controls, PSP and EOAD
Age at onset	66.19 ± 8.01	67.11 ± 7.77	70.60 ± 9.62*	74.28 ± 7.05*	57.26 ± 4.50*		*p* < 0.05; all cohorts (except DCB with PSP)
Age at death	76.10 ± 7.04	76.01 ± 8.58	80.24 ± 9.28*	83.40 ± 7.04*	67.89 ± 6.09*	NA	*p* < 0.05; all cohorts (except DCB with PSP)
Disease duration	10.12 ± 7.54	8.21 ± 3.91	9.67 ± 4.97	9.36 ± 4.96	10.79 ± 4.86*		*p* < 0.05; EOAD versus LOAD and PSP

*Note*: Data presented as mean ± SD or *N* (%).

Abbreviations: AD, Alzheimer's disease; CBD, corticobasal degeneration; EOAD, early onset AD; LOAD, late onset AD; NA, not available; PSP, progressive supranuclear palsy.

* Statistically significant differences among groups (*p* < 0.05).

Besides, males were diagnosed with AD earlier than females. (67.57 ± 10.04 years vs. 72.41 ± 8.91 years; *p* < 0.005) in AD, especially in LOAD (*p* < 0.005). Regarding the age at death, females (82.39 ± 8.45 years) have a higher age than males (76.51 ± 9.50 years; *p* < 0.005) in the AD, specifically in the LOAD (*p* < 0.005; 84.54 ± 6.47 female vs. 80.88 ± 7.60 male). Finally, women present a longer duration in AD (10.15 ± 4.76 years (female) vs. 8.86 ± 5.23 years (male); *p* < 0.005).

### 3.2 APOE in patients with tauopathies

As expected, the *APOE‐Ɛ4 all*ele frequency was higher in the AD cohort than in controls (52.3% vs. 18.1%, respectively; *p* < 0.001; OR [95% CI]: 4.96 [3.87‐6.38]) (Table S1). No other differences in the *APOE‐Ɛ4* allele frequency were observed when comparing other patient groups with controls. A different distribution of the Ɛ3Ɛ4 and Ɛ4Ɛ4 genotypes in AD, both LOAD and EOAD, was observed (Figure S2).

AD patients, particularly LOAD, with Ɛ4+ carriers had an earlier onset age (73.07 ± 6.56 years) and earlier mortality (82.35 ± 6.28 years) in comparison to Ɛ4 non‐carriers (onset: 75.67 ± 7.20 years; mortality: 84.73 ± 7.40 years; both *p* < 0.001). There were no differences in the distribution of APOE Ɛ4 genotype between genders in any cohort.

### 3.3 *HTT* pathological repeat expansions in patients with tauopathies

Two neuropathologically confirmed CBD (2/34 (5.9%)) and two neuropathologically confirmed PSP (2/98 (2.1%)) patients carried pathological CAG expanded *HTT* alleles. Two healthy female controls, aged 75 and 66 years‐old female healthy controls of the clinical cohort (0.19%; 2/1039) carried low penetrance‐pathological CAG expanded *HTT* alleles of 37 and 39 repeats, respectively. The frequency of expanded alleles was statistically different between CBD patients and controls (*p* = 0.0353; OR [95% CI]:31.20 [2.20‐438.26]). There was also a significant difference between PSP patients and controls (*p* = 0.037; OR [95% CI]: 11.20 [0.80–153.12]). One carrier of *HTT* alleles in the 36–39 range was observed among the AD group (0.2%), with a similar frequency to the control group (Table *2*). Loss of CAA interruptions was not detected after Sanger sequencing in the carriers of 36‐39 range CAG *HTT* alleles. The clinical features of carriers of the pathological CAG repeats are depicted in Table 3. None of them exhibited chorea, and there were no reported cases of HD in their family history.

**TABLE 2:** Frequencies of HTT IAs in the different cohorts.

Neuropathology	*N* genotyped	*HTT* IAs *N* (%)	*p*‐value (corrected)*	OR [CI 95%]	*HTT* pathological expansions *N* (%)	*p*‐value (corrected)*	OR [CI 95%]
CBD *n*=34	34	0 (0)	1.000	0.00 [0.00–3.16]	2 (5.9)	0.0353**	31.20 [2.20–438.26]
PSP *n* = 98	96	4 (4.2)	1.000	1.10 [0.28–3.13]	2 (2.1)	0.037***	11.20 [0.80–153.12]
AD *n* = 456	455	28 (6.2)	0.359	1.62 [0.95–2.73]	1 (0.2)	1.000	1.19 [0.02–22.90]
LO‐AD *n* = 363	362	24 (6.6)	0.404	1.76 [1.00–3.03]	1 (0.3)	1.000	1.50 [0.03–28.95]
EO‐AD *n* = 93	93	4 (4.3)	1.000	1.11 [0.28–3.17]	0 (0.0)	1.000	0.00 [0.00–60.95]
Controls *n* = 1070	1056	41 (3.9)			2 (0.2)		

Abbreviations: CI, confidence interval; OR, odds ratio.

* The threshold of significant *p*‐value is set below 0.05.

** HTT pathological expansions CBD, versus controls and AD.

*** HTT pathological expansions PSP versus controls. AD, Alzheimer's disease; CBD, corticobasal degeneration; EO‐AD, early onset AD; LO‐AD, late onset AD; PSP, progressive supranuclear palsy.

**TABLE 3:** Neuropathology and clinical data of the patients with expanded *HTT* CAG repeats.

	Case 1	Case 2	Case 3	Case 4	Case 5
Neuropathological diagnosis	PSP	PSP	CBD	CBD	AD
Comorbid pathology	Low AD‐NC (A1B1C0) Mild leptomeningeal CAA LBD (Braak 2)	LBD (Braak 1)	Intermediate AD‐NC (A3B2C3) Mild CAA AGD (Saito stage II) Moderate CVD	Mild amyloid plaque pathology (Thal stage 1) AGD (Saito stage II)	Moderate to severe CVD
Age at death(years)	78	73	75	75	96
Age at onset (years)	66	65	70	69	84
Clinical diagnosis	PSP vs. CBS	PSP	AD	FTD	AD
Main symptom	Gait disorder, supranuclear upward gaze palsy	Cognitive impairment, falls, non‐fluent aphasia, and vertical gaze palsy	Cognitive impairment, depression and irritability.	Cognitive impairment, with language and behavioural disorder	Cognitive impairment
Other signs/symptoms	Dystonia, cognitive impairment, dysarthria, dysphagia	Dysarthria	Other behavioural symptoms (obsessions, perseverance, aggressiveness, disinhibition, hallucinations, and delusions).	Aphasia and apraxia	‐
Chorea	No	No	No	No	No
Non‐motor symptoms	Depression, urinary incontinence	Multiple fainting episodes	‐	Behavioural impairment	‐
Other medical features	Hypertension, cholecystectomy, depressive syndrome	No data	Diabetes, hypertension, dyslipidaemia.	No data	No data
Neurological family history	Mother dementia	Paternal aunt dementia	Dementia, multiple sclerosis and Parkinson's disease	NO	No data
Familial Huntington disease	No	No	No	No	No data
Neuroradiology	MRI: few ischemic white matter lesions, no basal ganglia abnormalities, and age‐congruent global cortical atrophy	CT scan: bilateral frontal atrophy.	MRI: Global cortical atrophy with frontotemporal predominance SPECT: right frontal hypoperfusion, mild bilateral perisylvian hypoperfusion	No data	No data
*HTT* gene CAG repeats	27/40	17/37	19/36	20/36	17/36

Abbreviations: AD‐NC, Alzheimer's disease neuropathologic change; AGD, argyrophilic grain disease; CAA, cerebral amyloid angiopathy; CBD, corticobasal degeneration; CBS, corticobasal syndrome; CT, computed tomography; CVD, cerebrovascular disease; LBD, Lewy Body disease; MRI, magnetic resonance imaging; N/A, not available; PSP, progressive supranuclear palsy; SPECT, single photon emission computed tomography.

Additionally, we examined somatic instability across multiple brain regions obtained from the postmortem tissues (putamen, caudate, and cerebral cortex) of all patients with pathological expansion. No variation in the number of CAG repeats was observed in either case (Figure S3).

### 3.4 *HTT* IAs in tauopathies

AD, PSP, and CBD neuropathological groups showed no differences in the frequency of IAs compared with controls. Nevertheless, in the AD clinical group, the frequency of IAs carriers was slightly higher than that of controls (6.2% vs. 3.9%; *p* = 0.358; OR [95% CI]:1.62 [0.95–2.73]) (Table 2). When we stratified the AD patients into LOAD and EOAD, the IAs frequency was higher in LOAD (6.6%). However, this difference was not statistically significant compared to what was observed in EOAD (4.3%) or controls (3.9%).

No differences in the mean age at onset, age at death, disease duration, *APOE* isoforms, and gender between carriers and non‐carriers of IAs were observed in any of the groups when performing a multivariate logistic regression model. No association was confirmed performing an independent analysis of each variable.

### 3.5 Non‐pathological *HTT* CAG repeats in tauopathies

Considering the *HTT* size, AD patients (20.37 ± 3.41; *p* < 0.001; [95% CI]: [0.32–1.09]) had a higher mean of repeats in the longer *HTT* allele (allele 2) than controls (19.66 ± 3.39). A similar effect was observed for the short *HTT* allele, AD (16.80 ± 2.00; *p* < 0.001; [95% CI]: [−0.11 to 0.35]) compared with controls (16.68 ± 2.08). When we stratified the AD group, the LOAD group carried higher mean number of repeats in both larger and shorter alleles than controls (20.44 ± 3.44; *p* < 0.001; [95% CI]: [0.36–1.19]; 16.87 ± 1.89; *p* < 0.001; [95% CI]: [−0.06 to 0.43], respectively).

Multivariate logistic models were used to identify any independent factors related to the risk of tauopathies. The first model (Cox–Snell's *R* ^2^ = 0.0084), which included the number of CAG repeats in *HTT*, alleles 1 (shorter allele) and 2 (longer allele), revealed an association between the size of the *HTT* CAG allele 2 size and the development of LOAD (*β* = 0.069; *p* < 0.01). The second model (Cox–Snell's *R* ^2^ = 0.11) which included the size of *HTT* CAG alleles 1 and 2, *APOE* status (*APOE‐Ɛ4* carriers vs. non‐carriers; *APOE‐Ɛ2* carriers vs. non‐carriers) and gender, revealed that being female (*β* =0.695; *p* < 0.001), *APOE‐Ɛ4* (*β* = 1.33; *p* < 0.001), *APOE‐Ɛ2* (*β* = −1.05; *p* < 0.001) and *HTT* allele 2 size (*β* = 0.067; *p* < 0.01) are risk factors for LOAD. Furthermore, this second model showed that being female (*β* = −0.63; *p* < 0.005), *APOE‐Ɛ4* (*β* = 1.25; *p* < 0.001), and *APOE‐Ɛ2* (*β* = −1.52; *p* < 0.05) influence the development of EOAD (Cox–Snell's *R* ^2^ = 0.034). Also, we observed an additional effect of being female (*β* = −0.67; *p* < 0.005) and *APOE‐Ɛ4* (*β* = −0.99; *p* < 0.005) on the development of PSP (Cox–Snell's *R* ^2^ = 0.018). No other regression models displayed additional effects of the independent variables in the other groups.

Regarding age at onset, linear regression models were conducted, including the number of CAG repeats in *HTT* alleles 1 (shorter allele) and 2 (longer allele), but no significant associations were displayed. Cox survival studies were performed using the size of allele 1 and 2 as predictor variables, and no association between the size or number of CAG repeats in *HTT* and any neuropathological disease groups was found in these models.

### 3.6 Clinical and pathological description of patients with tauopathies and pathological *CAG* repeats carriers in the *HTT* gene

#### 3.6.1 CASE 1 (CAG 27/40)

A 66‐year‐old woman presented with a progressive abnormal left‐hand posture, gait disorder with multiple falls, cognitive impairment and vertical gaze palsy. The clinical impression was atypical parkinsonism (PSP vs. CBD), and levodopa therapy was started without significant improvement. The disease progressed, with the patient needing a wheelchair and developing urinary incontinence, dysarthria, and dysphagia. She passed away at 78 yo due to unknown infectious complications.

*Autopsy findings*: The microscopic examination revealed a predominant 4R tauopathy (Kovacs 4/6 stage) consistent with PSP.

Additionally, in the striatum, tau revealed abundant granular fuzzy astrocytes, to a lesser extent, tufted astrocytes and coiled bodies, and occasional globose neurofibrillary tangles and threads. Polyglutamine staining showed frequent intranuclear inclusions in the putamen and moderate in the caudate. Moderate gliosis and neuronal loss corresponding to a Vonsattel stage 2 were observed.

#### 3.6.2 CASE 2 (CAG 17/37)

A 65‐year‐old woman presented with progressive cognitive impairment, a gait disorder with multiple unprovoked falls, dysarthria, nonfluent aphasia, and vertical gaze palsy. The patient was diagnosed with PSP, gradually becoming reliant on a wheelchair. She passed away at 73 years.

*Autopsy findings*: Microscopic findings were consistent with PSP (Kovacs 4/6).

Moreover, in the head of the caudate there were moderate astroglial inclusions (granular fuzzy and tufted astrocytes) and sparse neurofibrillary tangles and oligodendroglial coiled bodies were present, with a higher concentration in the internal capsule and less frequent in the rostral putamen. Nuclear polyglutamine inclusions were moderate in the striatum. Mild gliosis was observed without apparent atrophy or neuronal loss, suggesting a maximum Vonsattel stage 1 in striatum degeneration.

#### 3.6.3 CASE 3 (CAG 19/36)

Patient 3 had a familial history of AD, multiple sclerosis, and Parkinson's. At 70, she started mild cognitive impairment accompanied by depression and irritability. Her condition remained stable until 73, when she exhibited a progressive deterioration in cognition and started behavioral changes, hallucinations, and delusions, significantly impacting her daily activities. She received a diagnosis of Alzheimer's disease and passed away at 75 due to multiorgan failure.

*Autopsy findings*: The microscopic evaluation indicated a combination of pathologies with the primary pathology being a CBD 4R tauopathy. Concomitant pathologies included intermediate Alzheimer's disease‐related neuropathological changes (A3B2C3, Thal 4/5, Braak IV/VI, CERAD severe) with mild cerebral amyloid angiopathy, argyrophilic grain disease (Saito stage II/III) and moderate cerebrovascular disease.

The striatum exhibited mild atrophy, accompanied by mild neuronal loss and mild to moderate gliosis. Frequent astrocytic plaques were observed, and neuronal inclusions were more commonly found in ventral areas, displaying moderate to frequent neurofibrillary tangles and pre‐tangles with scattered grains. Interestingly, in this case, only isolated nuclei with polyglutamine inclusions were observed in the caudate and putamen. Overall, the striatum could be categorized as a Vonsattel stage 2.

#### 3.6.4 CASE 4 (CAG 20/36)

Patient 4 was assessed for the first time at a Neurology Clinic at 72 years of age due to language and speaking problems, followed by severe behavioral symptoms and obsessive ideas. On examination a one‐year progressive clinical history was disclosed that included cognitive decline with a frontal profile and aphasic, apraxic and behavioral components. A clinical diagnosis of probable frontotemporal dementia (FTD), behavioral variant, moderate stage (GDS 5) was made at that time. On follow‐up the patient became fully mutist, developed severe gait problems with postural instability. Thereafter she needed a wheelchair, became totally uncontinent and had to be fed through a nasogastric tube. The patient died 6 years after clinical onset due to a respiratory infection.

*Autopsy findings*: On macroscopic examination, her brain (weight = 940 g) showed moderate to severe cortical atrophy with a frontotemporal distribution. Coronal sections of the left hemisphere (the right hemibrain was frozen following the brain bank protocol) revealed a moderate dilatation of the lateral ventricle and mild atrophy of the medial temporal lobe. On transversal sections of the brainstem, the substantia nigra was depigmented in its rostral segment. On H/E histological examination, severe cortical frontotemporal cortical atrophy was evidenced, with abundant ballooned cells. Phospho‐tau immunohistochemichal stains revealed frequent pretangles, Pick‐like bodies and astrocytic plaques at all cortical level examined. Medial temporal lobe structures displayed the same histological pictures, with the presence of argyrophilic grains in the entorhinal cortex. No inclusions immunoreactive to either α‐synuclein or TDP‐43 were observed at cortical or subcortical levels. Among subcortical structures, most relevant findings were observed in the substantia nigra, that showed intense neuronal loss and gliosis with frequent ballooned neurons. The final neuropathological diagnosis was sporadic primary tauopathy with a phenotypic profile of corticobasal degeneration.

#### 3.6.5 CASE 5 (CAG 17/36)

We have little information regarding case 5. At the age of 84 started with progressive cognitive impairment and was diagnosed with AD. She passed away at 93 yo due to infectious complications.

**FIGURE 2:**
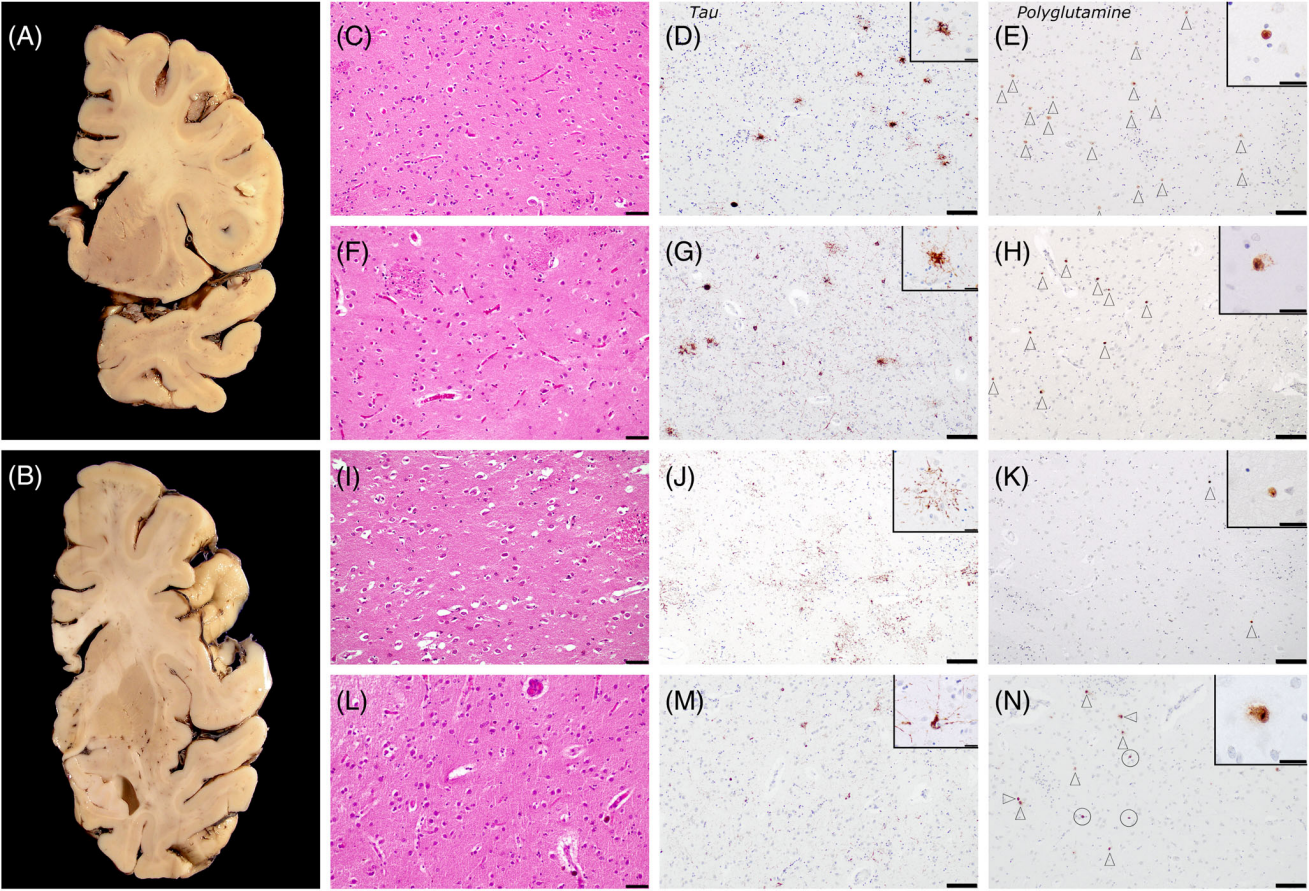
Neuropathological analysis of CAG HTT pathological expansion carriers A and B coronal sections of patient BK1291. Her brain weighed 1,105 grams, and macroscopic evaluation highlighted a mild to moderate temporoparietal cortical atrophy with ventricular dilation. The basal ganglia, including head of the caudate, were relatively spared, with minimal atrophy. Head of the caudate of patient BK393 in C to E, BK311 in F to H, BK1291 in I to K and BK654 in L to N. Hematoxylin and eosin showed moderate gliosis, with mild atrophy and neuronal loss in patient BK393 (C). There was mild parenchymal arteriosclerosis with mild perivascular rarefaction. Patient BK311 had mild gliosis without discernible atrophy or neuronal loss (F). In BK1291 (I) the head of the caudate and the rostral putamen nucleus showed mild atrophy, with mild neuronal loss and mild to moderate gliosis. They had mild arteriosclerosis with occasional perivascular hemosiderin deposits. BK654 (L) had a moderate parenchymal arteriosclerosis and arteriolohyalinosis, with perivascular rarefaction and hemosiderin deposits. The striatum showed mild to moderate gliosis (more intense in putamen), with minimal atrophy and neuronal loss. Staining against hyperphosphorylated Tau revealed moderate involvement of the caudate nucleus in patients with PSP (patients BK393 (D) and BK311 (G), insets tufted astrocytes), and in the patient with CBD (BK1291, inset astrocytic plaque). In patient BK645, with AD, Tau showed isolated neurofibrillary tangles and pretangles (M, inset neuronal pretangle). Polyglutamine positive neurons were detected in the caudate of all four patients (E, H, K, N, insets neuronal intranuclear polyglutamine inclusions), in variable densities (white arrowheads). Of note, BK1291 had comparatively more inclusions in parahippocampal cortex (not shown) compared to the head of the caudate (K), while in BK654 they could be confounded with moderate hemosiderin deposits (N, white circles). Scale bars: C, F, I, L 50 μm; D‐E, G‐H, J‐K‐M‐N 100 μm; Insets 20 μm.

*Autopsy findings*: Neuropathological findings were consistent with AD stage A3B3C3 (Thal phase 4/5 Braak stage V/VI CERAD frequent), along with comorbid moderate to severe CVD. Mild to moderate gliosis was observed in the striatum, along with minimal atrophy and neuronal loss. Moderate polyglutamine intranuclear inclusions were observed in caudate and putamen. Amyloid beta staining revealed extensive diffuse plaques, while tau staining demonstrated isolated neurofibrillary tangles and pre‐tangles. The observed histological changes in the striatum indicated a Vonsattel stage 2 (Figure 2; File S1).

### 3.7 Presence and distribution pattern of polyglutamine inclusions in individuals with tauopathies and pathological expansions of *HTT*

Next, we studied whether patients with tauopathies and pathological expansions of *HTT* had a differential distribution pattern based on the subtype of tauopathy. To achieve this, sections from crucial brain regions—frontal cortex, striatum globus pallidus, hippocampus, midbrain, and cerebellum—were analyzed in cases 1–3 and 5, encompassing four HD cases and six subjects diagnosed with tauopathy but lacking pathological *HTT* expansions (PSP *n* = 2, CBD *n* = 2, and AD *n* = 2). The analysis was conducted in a blind manner, with no knowledge of the diagnosis or the number of *HTT* CAG repeats.

In cases 1–3, we observed that the density of aggregates stained with polyglutamine was higher in subjects with more CAG pathological expansions in the *HTT* gene. Case 1, who had the highest number of pathological expansions and is also a carrier of an IA (CAG 27/40), showed a higher frequency of intranuclear inclusions than subject 2, with 17/37 CAG repeats, who had a mild to moderate frequency, or subject 3, with 19/36 repeats and isolated inclusions. However, case 5, who had the lowest number of repetitions (CAG 16/36) among all patients, had the second‐highest number of inclusions, suggesting that in these cases, other factors beyond the number of repeats could influence the accumulation of these polyglutamine peptides.

Regarding the distribution of polyglutamine inclusions, subjects with atypical parkinsonism (PSP and CBD) showed more involvement in the frontal, occipitotemporal, and striatal regions than in other regions, such as the subiculum or the parahippocampal gyrus. On the contrary, subject 5, with AD, had the highest number of inclusions in the parahippocampal gyrus. In HD, polyglutamine accumulations were frequent in the frontal cortex, parahippocampal gyrus, occipital cortex, and putamen; moderate to frequent in the nucleus basalis of Meynert, caudate, and substantia nigra; moderate in the subiculum and midbrain tegmentum; mild in the dentate nucleus; and isolated in the cerebellar cortex. Unlike HD cases, patients with tauopathies and pathological CAG repeats did not show cerebellar inclusions, and mesencephalic involvement was scarce in one patient. It is worth noting that none of the subjects in the control group, consisting of individuals with tauopathies and normal CAG repeat numbers in the *HTT* gene, showed any inclusions or nuclear immunoreactivity with polyglutamine IHC in any of the studied regions (Figure 2, Table 4).

**TABLE 4:** Presence and distribution pattern of polyglutamine inclusions in individuals with tauopathies and pathological expansions in the *HTT* gene.

	FxCx	SB	PhG	OTCx	MBN	GP	Putamen	Caudate	SN	MbTg	Dent Nuc	Cb
Case 1	*3+*	*2+*	*2+*	*2+*	*1+*	*0.5*	*3+*	*2+*	*0.5*	*0*	*0*	0
Case 2	*1+*	*0.5*	*1*	‐	*2+*	*0.5*	*2+*	*2+*	‐	‐	*0*	0
Case 3	*0.5*	*0*	*0*	*1+*	*0.5*	*0*	*1+*	*1+*	*0*	*0*	*0*	0
Case 5	*2+*	*1+*	*3+*	*2+*	*2+*	*0*	*2+*	*2+*	*0*	*0*	*0*	0
HD1	*3+*	*2+*	*3+*	*3+*	*3+*	*2+*	*3+*	*3+*	*3+*	*2+*	*1+*	0.5
HD2	*3+*	*2+*	*3+*	*3+*	*3+*	*0.5*	*3+*	*3+*	*2+*	*2+*	*2+*	0.5
HD3	*3+*	*2+*	*3+*	*3+*	*3+*	*0.5*	*3+*	*2+*	*3+*	*2+*	*2+*	0.5
HD4	*3+*	*2+*	*3+*	*3+*	*2+*	*1+*	*3+*	*3+*	*2+*	*1+*	*1+*	0.5
PSP1	0	0	0	0	0	0	0	0	0	0	0	0
PSP2	0	0	0	0	0	0	0	0	0	0	0	0
CBD1	0	0	0	0	0	0	0	0	0	0	0	0
CBD2	0	0	0	0	0	0	0	0	0	0	0	0
AD1	0	0	0	0	0	0	0	0	0	0	0	0
AD2	0	0	0	0	0	0	0	0	0	0	0	0

*Note:* Frequent (3+): Indicates a high prevalence or severity of polyglutamine inclusions in the respective brain region. Moderate (2+): Suggests a moderate prevalence or severity of polyglutamine inclusions. Mild (1+): Implies a mild presence or severity of polyglutamine inclusions. Isolated (0.5): Indicates sporadic or isolated instances of polyglutamine inclusions. None (0): Denotes the absence of polyglutamine inclusions in the respective brain region.

Abbreviations: AD, Alzheimer's disease; Cb, cerebellum; CBD, corticobasal degeneration; Dent Nuc, dentate nucleus; FxCx, frontal cortex; GP, globus pallidus; HD, Huntington's disease; MBN, Meynert's basal nucleus; MbTg, midbrain tegmentum; OTCx, occipitotemporal cortex; PhG, parahippocampal gyrus; Polyglutamine inclusions, frequent (3+), moderate (2+), mild (1+), isolated (0.5), and none (0); PSP, progressive supranuclear palsy; SB, subiculum; SN, substatia nigra.

## 4 DISCUSSION

In this study, we investigated whether CAG repeats in the *HTT* gene can lead to a risk of developing tauopathies and we performed a neuropathological analysis in pathological expansion and IAS *HTT* carriers in order to detect polyglutamine aggregates. We identified two CBD patients (5.9%), two PSP patients (2.1%), and one AD patient (0.2%) who carried pathogenic *HTT* CAG repeats. Four out of five patients are carriers of low penetrance *HTT* repeat expansions (36 and 37 repeats). The fifth subject was a PSP patient who carried an IA (27 CAG repeats) and a full penetrance *HTT* repeat expansion (40 CAG repeats). We found two healthy controls from the clinical cohort, also carriers of low penetrance *HTT* CAG expansions (0.19%), similar to what has been published giving consistency to our results [23]. The differences in frequency distribution were statistically significant in the CBD and PSP groups compared to the healthy controls.

Many studies have emphasized the significance of interruption CAA loss as an independent contributing factor to an earlier age of onset of the HD disease, especially in individuals with reduced penetrance mutations and increased somatic repeat instability [24, 25, 26]. We examined the size of CAG repeats in four brain regions (cerebellum, putamen, caudate and cerebral cortex) of the pathological expansion carriers. These tissues display the most pronounced instability of CAG repeat in HD within the central nervous system [27]. The absence of somatic mosaicism might explain the clinical presentation observed in the four cases. In fact, none of the cases reported here showed interruption CAA loss.

In all pathological expansion carriers, none of them experienced choreic movements or reported family history of HD and no association between the length of CAG pathological repeats and the tauopathy phenotype was observed. Although we cannot definitively confirm whether certain symptoms, such as cognitive impairment or psychiatric disorders, could be attributed to HD, both the clinical presentation and the pathological findings left no doubt that they developed a tauopathy. Additionally, although acknowledging the limitation due to the study's retrospective nature, patients with PSP or DCB were examined by a well‐known movement disorder specialist and no atypical motor signs were listed in their respective medical records. It is worth emphasizing that individuals with HD may exhibit oculomotor alterations and unprovoked falls, but these manifestations tend to emerge at a more advanced stage rather than in initial stages as observed in our reported case.

Patients with HD are pathologically characterized by progressive neuronal loss and gliosis in the striatum, which can be assessed using the Vonsattel stages [28]. Thus, we aimed to evaluate whether the pathological expansion carriers had a greater degree of striatal atrophy than expected. We observed that they had mild striatal involvement (stages 1–2), which could be attributed to the underlying tauopathy, Nevertheless, considering the clinical context, an alternative explanation, particularly for the cases with a higher burden of CAG inclusions (Case 1 and Case 5), could be, that although they displayed subtle or mild signs associated with HD, it was difficult to distinguish from the prevailing clinical profile of tauopathies (PSP, CBD, or AD). Late‐onset HD diagnosis can be difficult due to milder symptoms and the elevated prevalence of neurodegenerative disorders in the older population [29]. Additionally, the diagnosis may be further complicated by the absence of a family history, which is very frequent in individuals with low‐penetrance gene mutations and late‐onset HD [30].

These results suggest an enrichment of the *HTT* expansion in 4R‐type tauopathies. In fact, it has recently been described that the pathogenic expansion within the *HTT* gene could manifest as a PSP phenotype [31]. Moreover, there is empirical evidence revealing an elevation in the 4R‐Tau/3R‐Tau ratio, at both the protein and mRNA levels, in putamen samples from individuals with HD, attributed to an up‐regulation of 4R‐Tau isoforms [8, 32]. Additionally, in our investigation, we conducted screenings for polyglutamine inclusions within our cohort of Pick's disease patients with 3R‐Tau isoforms (*n* = 13), and none of them displayed any inclusions (data not shown). Altogether, our study reinforces the relationship between *HTT* expansions and 4R tauopathies.

With regards to the analysis of *HTT* CAG IAs, we found an increased frequency of *HTT* IAs in the LOAD neuropathological group compared with controls (6.6% vs. 3.9%). This difference was not statistically significant perhaps due to the small sample of the LOAD cohort. However, the IAs increased frequency in LOAD confirms our previous result in an AD clinical cohort that suggested that CAG repeats in the *HTT* gene might play a role in the risk of the disease. In addition, we found an increase of *HTT* CAG repeat size in the LOAD group. It seems that their risk effect can be associated with gender and other genetic factors such as the *APOE4* allele. Important evidence supporting the association of the *HTT* gene with AD is the reported neuropathological study of 15 elderly HD subjects that found evidence of co‐occurring AD neuropathology in 82% of the cases with prominent dementia [33]. Recently, a neuropathological study suggested that *HTT* accumulates in pyramidal neuron‐rich areas including hippocampal subregions associated with memory and frontal cortex layer III [4]. Thus, the role of *HTT* as a modulator of AD neurodegeneration could be possible.

In the last few years, several studies assessed the potential pathogenic effect of *HTT* CAG repeats in several genes on different neurodegenerative diseases. Expansions of the CAG repeats in the *ATXN2* gene have been associated with increased risk of ALS and could be a FTD phenotype modifier and intermediate *ATXN1* alleles have been proposed as a risk factor in ALS [34]. In a previous multicentric study, we have identified a significant association of *HTT* IAs with increased disease risk for FTD and a significant increase of *ATXN2* IAs in AD patients. In addition, we also found an increased frequency of *HTT* IAS in behavioral FTD variant (bvFTD) and PNFA [13].

A recent study in FTD/ALS and synucleinopathies showed a frequency of low‐penetrance (36–39) *HTT* repeat expansions of 0.20, 0.19%, 0.3% in FTD/ALS, LBD, PD, respectively, like that the observed in healthy controls (0.22%) suggesting that low‐penetrance *HTT* repeat expansions have no effect in TDP‐43 or α‐synuclein proteinopathies. However, IAs frequency was increased in the multiple system atrophy (MSA) and MSA carriers with 32 *HTT* CAG repeats showed isolated polyglutamineQ inclusions in pons and basal nuclei, which are two critical structures in the neurodegeneration of MSA. Besides it had demonstrated the presence of huntingtin‐positive aggregates mainly in the frontal cortex of frontotemporal lobar degeneration (FTLD)‐TDP43 patients with pathological expansions of *the HTT* gene [11, 14].

We investigated whether the distribution of polyglutamine inclusions varied based on the tau phenotype in individuals with pathological expansions of the *HTT* gene. From the explored regions (frontal cortex, striatum, globus pallidus, hippocampus, midbrain, and cerebellum), we observed that in subjects with atypical parkinsonism, the frontal cortex and striatum were more prominently affected than in the AD patient which the parahippocampal involvement was more pronounced. This adds further evidence that there might be a differential distribution based on the most affected regions in different tauopathies. However, the absence of polyglutamine inclusions in a relevant region for PSP such as the midbrain tegmentum, which does accumulate inclusions in HD, suggests that perhaps not all relevant regions would act in a similar manner. Nevertheless, considering the limited number of subjects analyzed, further studies would be necessary to explore the relationship between both pathologies.

All these data suggest that *HTT* CAG repeats might play a wide role in a small subset of cases with different neurodegenerative diseases. Many neurodegenerative diseases accumulate different protein aggregates in the brain, such as amyloid, tau, alpha‐synuclein, or TDP43. Furthermore, very often different protein aggregates coexist within the same brain [32]. The brain regions initially affected by these aggregates and the clinical symptoms are also different among the different neurodegenerative conditions. There is increasing evidence for the presence of pathological forms of tau in tissues of both HD patients and animal models and many data support the interactions between mutant Htt and other pathological proteins. Thus, it has been shown that Htt interacts with tau kinases, phosphatases and other proteins, as serine/arginine‐rich splicing factor 6 (SRSF6), which participate in tau alternative splicing. Moreover, mutant Htt plays a role in calcineurin dysregulation which triggers hyperphosphorylation of tau and it is recruited by tau to the microtubule network causing disruption and alteration of axonal transport [35, 36].

## 5 CONCLUSIONS AND LIMITATIONS

Our results, in line with previous works, pointed to a link between *HTT* CAG repeats and other non‐HD brain proteinopathies and support the hypothesis that they can share common pathways. Considering the clinical‐pathological heterogeneity of tauopathies, one of the strengths of this study is that it was conducted with confirmed neuropathological samples of PSP, CBD, and AD. However, we are aware that our study has several limitations, since the sample size for some of neuropathological cohorts was small, suggesting the need for replication of additional series to confirm these results. The retrospective nature has precluded detailed clinic‐pathological correlation in some cases. Although the possibility of stochastic association is minimized by the significant difference with controls, still it cannot completely be ruled out, particularly since the neuropathological cohort of controls is small compared both to the clinical cohort of controls and to the neuropathological cases with tauopathy.

Finally, there is a growing interest in investigating the presence of concomitant pathology in neurodegenerative diseases, especially in order to optimize the treatment when new effective drugs become available. Given that expansions of HTT have been associated with a different proteinopathies and considering the evidence of increased aggregation of phosphorylated Tau, α‐Syn, and TDP‐43, mostly in later stages of HD, we could hypothesize that patients with pathological expansions may have a greater number of copathologies. Although, in our study the identified cases do not show a significant number of proteinopathies, new studies must be necessary to delve deeper into this aspect.

## AUTHOR CONTRIBUTIONS

**Sergio Pérez‐Oliveira**: Analyzed and interpreted the data; drafted the manuscript for intellectual content. **Juan Castilla‐Silgado**: Analyzed and interpreted the data; drafted the manuscript for intellectual content. **Cèlia Painous**: Analyzed and interpreted the data; drafted the manuscript for intellectual content. **Iban Aldecoa**: Execution (Biobank data). Critical revision of the manuscript. **Manuel Menéndez‐González**: Execution (clinical data). Critical revision of the manuscript. **Marta Blázquez‐Estrada**: Execution (clinical data). Critical revision of the manuscript. **Daniela Corte**: Execution (Biobank tissues). Critical revision of the manuscript. **Cristina Tomás‐Zapico**: Execution (genetic data). Critical revision of the manuscript. **Yaroslau Compta**: Execution (clinical data). Critical revision of the manuscript. **Esteban Muñoz**: Execution (clinical data). Critical revision of the manuscript. **Albert Lladó**: Execution (clinical data). Critical revision of the manuscript. **Mircea Balasa**: Execution (clinical data). Critical revision of the manuscript. **Gemma Aragonès**: Execution (Biobank). Critical revision of the manuscript. **Pablo García‐González**: Execution (control samples). Critical revision of the manuscript. **Maitée Rosende‐Roca**: Execution (control samples). Critical revision of the manuscript. **Mercè Boada**: Execution (control samples). Critical revision of the manuscript. **Agustín Ruíz**: Execution (control samples). Critical revision of the manuscript. **Pau Pastor**: Execution (clinical data). Critical revision of the manuscript. **Beatriz De la Casa‐Fages**: Execution (clinical data). Critical revision of the manuscript. **Alberto Rabano**: Execution (clinical data). Critical revision of the manuscript. **Raquel Sánchez‐Valle**: Execution (clinical data). Critical revision of the manuscript. **Laura Molina‐Porcel**: Design and conceptualized study; interpreted the data. Execution (neuropathology) of the project, statistical analysis (design and critical analysis) and writing of the first draft of the manuscript. **Victoria Álvarez**: Design and conceptualized study; interpreted the data. Execution (genetics) of the project, statistical analysis (design and critical analysis) and writing of the first draft of the manuscript.

## FUNDING INFORMATION

This study has been funded by Instituto de Salud Carlos III (ISCIII) and cofunded by European Union through the project P21/00467 and co‐funded by the European Union. Sergio Pérez‐Oliveira is supported by Asociación Parkinson Asturias‐Obra Social Cajastur and by Fundación para la Investigación y la Innovación Biosanitaria del Principado de Asturias (FINBA). Juan Castilla‐Silgado is supported by a contract associated with grant AC20/00017 from Instituto de Salud Carlos III, Spain, cofunded by EuroNanoMed III (grant 20‐0084). The Genome Research @ Ace Alzheimer Center Barcelona project (GR@ACE) is supported by Grifols SA, Fundación bancaria ‘La Caixa’, Ace Alzheimer Center Barcelona ‐Universitat Internacional de Catalunya. Ace Alzheimer Center Barcelona is one of the participating centers of the Dementia Genetics Spanish Consortium (DEGESCO). Agustín Ruíz and Mercé Boada receive support from the European Union/EFPIA Innovative Medicines Initiative joint undertaking ADAPTED and MOPEAD projects (grant numbers 115975 and 115985, respectively). Mercé Boada and Agustín Ruíz are supported by Instituto de Salud Carlos III ( ISCIII) and FEDER PI13/02434, PI16/01861, PI17/01474, PI19/01240, PI19/01301, and PI22/01403. Acción Estratégica en Salud is integrated into the Spanish National R + D + I Plan and funded by ISCIII (Instituto de Salud Carlos III)‐Subdirección General de Evaluación and the Fondo Europeo de Desarrollo Regional (FEDER‐“Una manera de Hacer Europa”). Pablo García‐González is supported by CIBERNED employment plan CNV‐304‐PRF‐866. Agustín Ruíz is supported by ISCIII national grant PMP22/00022, funded by the European Union (NextGenerationEU).

## CONFLICT OF INTEREST STATEMENT

The authors report no conflicts of interest related to this work.

## ETHICS STATEMENT

We obtained the approval of Ethical Committees of Neurological Tissue Bank (NTB) of the Hospital Clinic‐FRCB‐IDIBAPS, Barcelona, Spain, the Principado de Asturias BioBank, Biobank Banco de Tejidos Fundación CIEN and the Ethical Committees of Hospital Universitario Central de Asturias and FUNDACIÖN ACE (Barcelona).

## CONSENT FOR PUBLICATION

No applicable.

## Supporting information


**Figure S1.** Representative TP‐PCR electropherograms showing HTT normal, intermediate and expanded CAG alleles.

**Figure S2.** Distribución de APOE genotypes across the sample. BD, corticobasal degeneration; PSP, progressive supranuclear palsy; LO‐AD, late‐onset Alzheimer Disease; EO, early‐onset Alzheimer Disease; AD, Alzheimer disease; Controls, healthy controls.

**Figure S3.** Analysis of Somatic instability between brain tissues. Capillary electrophoresis of those patients with pathogenic expansions of the CAG HTT repeats. Tissues of the prefrontal cortex, caudate, putamen, and cerebellum were compared. CBD, corticobasal degeneration; AGD, argyrophilic grain disease; PSP, progressive supranuclear palsy; AD, Alzheimer's disease.

**Table S1.** APOE Ɛ4 and Ɛ2 carriers in the different cohorts.

**Data S1:** Supporting Information.

## Data Availability

The raw dataset generated in the current study are available from the corresponding author upon reasonable request.
