# Supplementary figures and images for: Huntingtin CAG repeats in neuropathologically confirmed tauopathies: Novel insights
Source: Brain Pathol. 2024 Feb 28;34(4):e13250. doi: 10.1111/bpa.13250 (PMC11189778; doi:10.1111/bpa.13250)

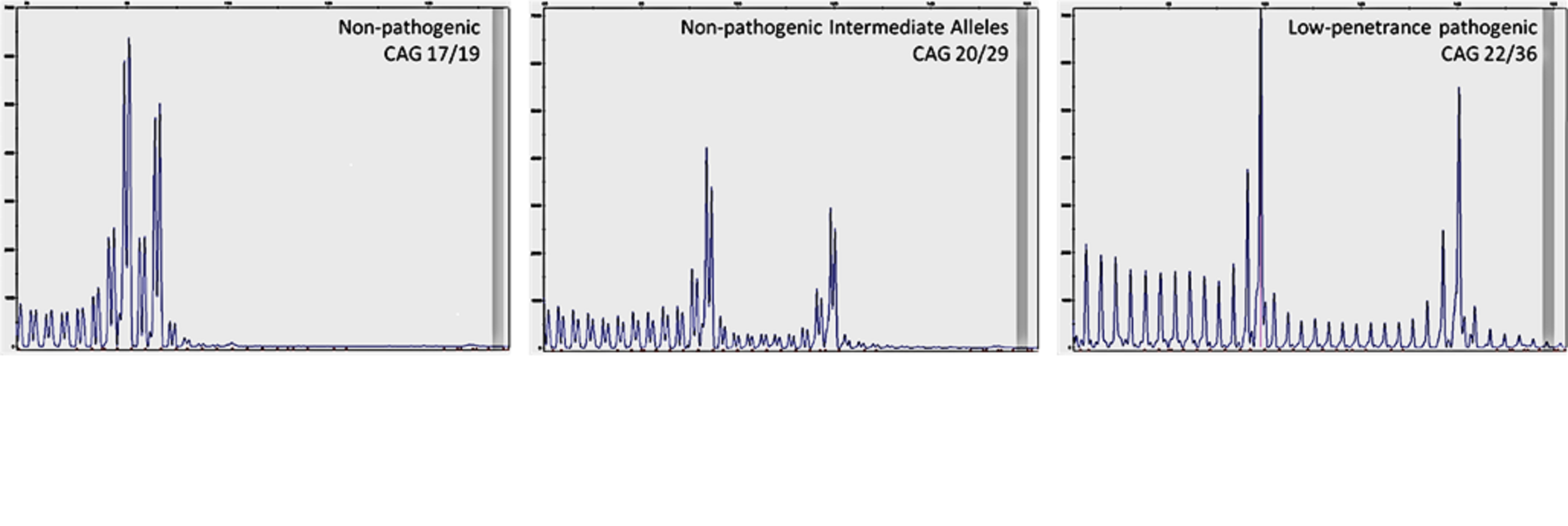

Supplement: Supplementary file 1 — Figure S1. Representative TP‐PCR electropherograms showing HTT normal, intermediate and expanded CAG alleles. [file BPA-34-e13250-s005.tif]

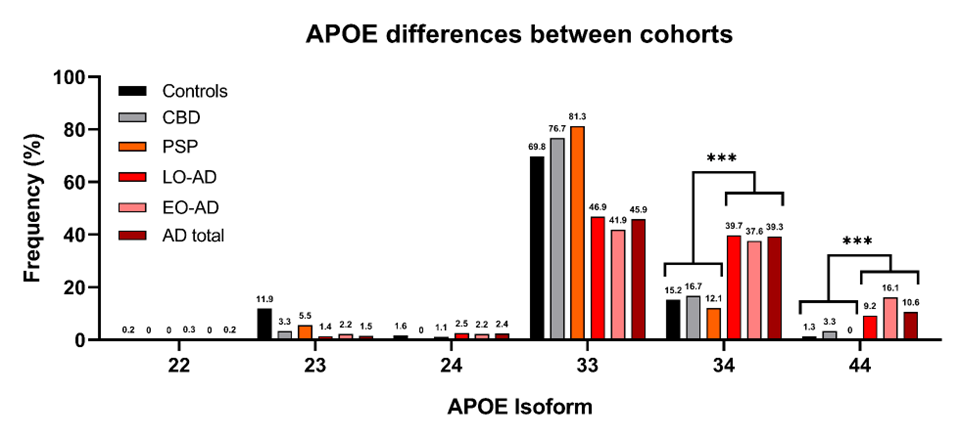

Supplement: Supplementary file 2 — Figure S2. Distribución de APOE genotypes across the sample. BD, corticobasal degeneration; PSP, progressive supranuclear palsy; LO‐AD, late‐onset Alzheimer Disease; EO, early‐onset Alzheimer Disease; AD, Alzheimer disease; Controls, healthy controls. [file BPA-34-e13250-s004.tif]

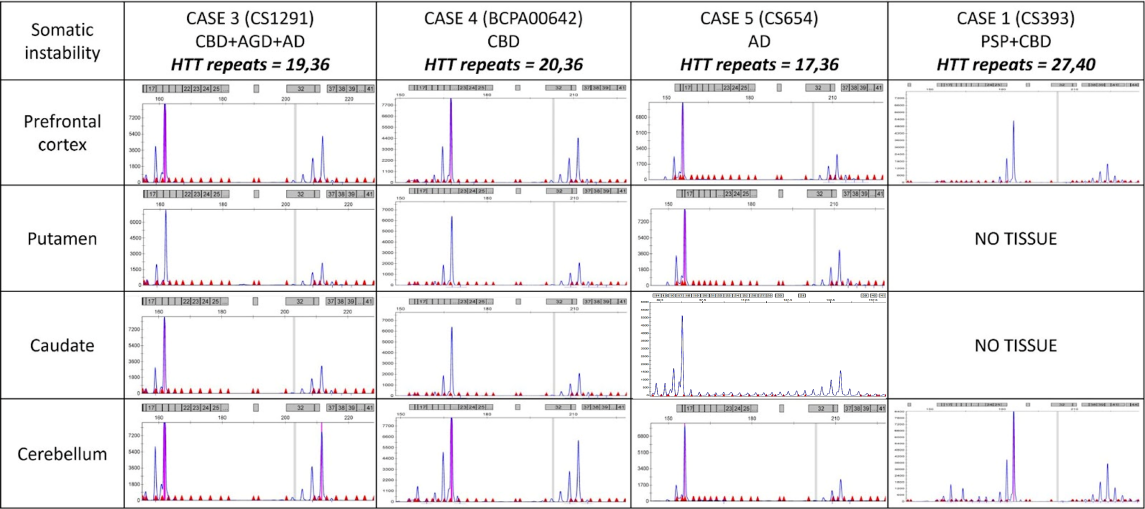

Supplement: Supplementary file 3 — Figure S3. Analysis of Somatic instability between brain tissues. Capillary electrophoresis of those patients with pathogenic expansions of the CAG HTT repeats. Tissues of the prefrontal cortex, caudate, putamen, and cerebellum were compared. CBD, corticobasal degeneration; AGD, argyrophilic grain disease; PSP, progressive supranuclear palsy; AD, Alzheimer's disease. [file BPA-34-e13250-s002.tif]
