# Supplementary material for: Huntingtin CAG repeats in neuropathologically confirmed tauopathies: Novel insights
Source: Brain Pathol. 2024 Feb 28;34(4):e13250. doi: 10.1111/bpa.13250 (PMC11189778; doi:10.1111/bpa.13250)
Supplement: Supplementary file 4 — Table S1. APOE Ɛ4 and Ɛ2 carriers in the different cohorts. [file BPA-34-e13250-s001.docx]

**Supplementary table 1. APOE Ɛ4 and Ɛ2 carriers in the different cohorts.**

|  | Samples genotyped ( N) | **APO*Ɛ4+*** | | | **APO*Ɛ*2+** | | |
| --- | --- | --- | --- | --- | --- | --- | --- |
|  |  | N (%) | p-value corrected | OR [CI 95%] | N (%) | p-value corrected | OR [CI 95%] |
| **CBD**  **n=34** | 33 | 6 (18.2) | 1.0 | 1.01 [0.33-2.53] | 4 (12.1) | 1.0 | 0.86 [0.22-2.51] |
| **PSP**  **n=98** | 91 | 12 (13.2) | 1.0 | 0.69 [0.33-1.30] | 6 (6.6) | 0.422 | 0.44 [0.16-1.03] |
| **AD**  **n=456** | 453 | 237 (52.3) | **1.06e-38*** | 4.96 [3.87-6.38] | 18 (4.0) | **1.22e-8*** | 0.26 [0.15-0.43] |
| **LO-AD**  **n=363** | 360 | 185 (51.4) | **1.92e-31*** | 4.78 [3.66-6.26] | 15 (4.2) | **1.16e-6*** | 0.27 [0.15-0.47] |
| **EO-AD**  **n=93** | 93 | 52 (55.9) | **1.21e-13*** | 5.73 [3.61-9.13] | 3 (3.2) | **0.0166*** | 0.21 [0.04-0.65] |
| **Controls**  **n=1070** | 1039 | 188 (18.1) |  |  | 143 (13.8) |  |  |

*^*Significantly different among groups (p < .05). OR: Odds ratio; CI: Confidence interval. CBD:Corticobasal degeneration; PSP: Progressive supranuclear palsy; AD: Alzheimer’s Disease; LO-AD: Late onset AD; EO-AD: Early onset AD.^*
