# Supplementary material for: Huntingtin CAG repeats in neuropathologically confirmed tauopathies: Novel insights
Source: Brain Pathol. 2024 Feb 28;34(4):e13250. doi: 10.1111/bpa.13250 (PMC11189778; doi:10.1111/bpa.13250)
Supplement: Supplementary file 5 — Data S1: Supporting Information. [file BPA-34-e13250-s003.docx]

**SUPPLEMENTARY AUTOPSY DESCRIPTION**

***CASE 1 (CAG 27/40)***

The brain weighed 1,180g and showed frontal cortical atrophy and mild depigmentation of the substantia nigra and locus coeruleus. The microscopic examination revealed moderate neuronal loss and gliosis in the frontal cortex, caudate, putamen, pallidum, substantia nigra and dentate nucleus, and to a lesser extent, the thalamus. Microscopical findings revealed a 4R tauopathy with moderate to severe involvement of subcortical areas, the brainstem, and the limbic system, and moderate involvement in the frontal cortex and cerebellum with minimal involvement in the occipital region (Kovacs 4/6 stage). Immunohistochemistry with AT8 demonstrated tufted and granular fuzzy astrocytes, oligodendroglial coiled bodies, pre-tangles, and neurofibrillary tangles with moderate to severe involvement of subcortical areas, including substantia nigra and mesencephalic tegmentum, as well as the limbic system. The frontal cortex, cerebellar dentate nucleus, and white matter showed moderate involvement.

Comorbid pathology consisted of incipient neurofibrillary pathology in the trans entorhinal cortex (Braak stage I/VI), isolated diffuse neocortical plaques (Thal phase 1/5, CERAD 0), minimal leptomeningeal cerebral amyloid angiopathy and mild Lewy body disease in the brainstem (Braak stage 2/6) with concomitant mild involvement of the olfactory bulb and tract. No TDP43 inclusions were identified.

In the striatum, tau revealed abundant granular fuzzy astrocytes and, to a lesser extent, tufted astrocytes and coiled bodies. There were also occasional globose neurofibrillary tangles and threads. Polyglutamine staining showed frequent intranuclear inclusions in the putamen and moderate in the caudate. There was mild parenchymal arteriosclerosis with mild perivascular rarefaction. The moderate gliosis and neuronal loss observed were compatible with a degree of degeneration corresponding to Vonsattel stage 2.

***CASE 2 ( CAG 17/37)***

**Autopsy description**: the macroscopic description was unavailable. Microscopic findings were consistent with PSP. Neuronal loss and gliosis were moderate in the cortex, mild in the basal ganglia and hippocampus, and severe in the dentate nucleus and cerebellar white matter. The midbrain could not be evaluated. A predominant 4R-type tauopathy (Kovacs 4/6) with tufted astrocytes, granular fuzzy astrocytes, tangles, pre-tangles, and coiled bodies was observed with severe involvement in the dentate nuclei and moderate in the neocortex and basal ganglia.

Occasional Lewy bodies and neurites were observed in the dorsal motor nucleus of the vagus nerve and basal forebrain (Braak 1). No evidence of amyloid beta or TDP43 deposits was found.

There were moderate astroglial inclusions, specifically granular fuzzy astrocytes and tufted astrocytes in the head of the caudate. Additionally, sparse neurofibrillary tangles and oligodendroglial coiled bodies were also present, with a higher concentration observed in the internal capsule. However, the inclusions were less frequent in the rostral putamen. Nuclear polyglutamine inclusions were moderate in the striatum, but no evidence of alpha-synuclein pathology was detected. Mild gliosis was observed without apparent atrophy or neuronal loss, suggesting a maximum Vonsattel stage 1 in striatum degeneration.

***CASE 3 CAG 19/36)***

**Autopsy description:** The main neuropathologic diagnosis was CBD. The brain weighed 1,105 grams, and macroscopic evaluation revealed moderate temporoparietal cortical atrophy, mild medial temporal and vermian atrophy, and moderate pigment loss in the substantia nigra.

H&E staining revealed moderate neuronal loss with moderate astrogliosis in cortical areas. Dense eosinophilic extracellular plaques were detected. In the hippocampus, there was an abundance of neurons showing granulovacuolar degeneration. A prominent loss of pigmented neurons in the substantia nigra, particularly in the ventral region, was notable, accompanied by abundant free neuromelanin pigment. A mild loss of Purkinje cells in the cerebellar vermis with isolated axonal torpedoes was also observed.

The microscopic evaluation indicated a combination of pathologies. The primary pathology was a 4R tauopathy consistent with CBD. Astrocytic plaques, granular fuzzy astrocytes, oligodendroglial coiled bodies, pre-tangles, and neurofibrillary tangles were observed, with frequent involvement in the basal ganglia, moderate involvement in the frontal cortex and mild involvement in the substantia nigra and pons, without cerebellar involvement, consistent with stage 4/4 according to Kovacs criteria. Beta-crystallin immunostaining highlighted a moderate frequency of ballooned neurons in the neocortex. Concomitantly, there were prominent ballooned neurons in the cingulate and parietal cortex, along with coiled bodies and aggregates of hyperphosphorylated tau in glial cells in the form of astrocytic plaques in the frontal cortex, cingulate cortex, caudate, and putamen.

Alongside CBD, there were concomitant pathologies, including intermediate Alzheimer's disease-related neuropathological changes (A3B2C3, Thal 4/5, Braak IV/VI, CERAD severe) with mild cerebral amyloid angiopathy, argyrophilic grain disease (Saito stage II/III) was also present and moderate cerebrovascular disease with vascular brain injury (multiple lacunar infarcts in the basal ganglia and basis pontis). There was no evidence of alpha-synuclein or TDP43 deposits.

The striatum exhibited mild atrophy, accompanied by mild neuronal loss and mild to moderate gliosis. Frequent astrocytic plaques were observed, and neuronal inclusions were more commonly found in ventral areas, displaying moderate to frequent neurofibrillary tangles and pre-tangles with scattered grains. Abundant amyloid-beta deposits were present in the form of mature and primitive plaques, as well as cores, with diffuse deposits being less prominent. Additionally, mild arteriosclerosis was noted, along with occasional perivascular hemosiderin deposits. Interestingly, in this case, only isolated nuclei with polyglutamine inclusions were observed in the caudate and putamen. Overall, the striatum could be categorized as a Vonsattel stage 2 in terms of degeneration.

***CASE 5 (CAG 14/36)***

**Autopsy description:** The brain weighed 1.020g. Diffuse global atrophy was observed during the external examination, accompanied by significant ventricular dilation in coronal sections. The striatum displayed perivascular dilations and lacunar infarcts, with the largest measuring up to 16mm in the putamen. Focal areas of yellowish degeneration were present in the cerebellar white matter near the dentate nucleus, consistent with remote vascular brain injury foci.

H&E staining, in addition to the infarcts described in the macroscopic examination, revealed moderate to severe cerebrovascular disease characterized by moderate parenchymal arteriosclerosis and arterial hyalinosis. There was a marked hippocampal atrophy with moderate gliosis and mild neuronal loss. Moderate changes were observed in the neocortex, while the substantia nigra showed mild changes. Notably, there was neuronal loss in the dentate nucleus and moderate gliosis in the context of cerebrovascular pathology.

Upon neuropathological examination, abundant neocortical senile plaques were observed, as well as in the hippocampus, basal nuclei, and to a lesser extent, in the brainstem, including the pons, but not in the cerebellum. Numerous neurons showed neurofibrillary degeneration, along with plaques and neurofibrillary tangles in the neuropil of the transentorhinal cortex, entorhinal cortex, hippocampus, amygdala, cingulate cortex, Meynert nucleus, and sparsely in the neocortex. Similar pathology was observed in the brainstem, mainly in the substantia nigra, locus coeruleus, and raphe nuclei. Sparse amyloid deposits were seen in the walls of the cerebral leptomeningeal vessels. Findings were consistent with Alzheimer's disease-related neuropathologic changes, corresponding to stage A3B3C3 (Thal phase 4/5 Braak stage V/VI CERAD frequent).

The striatum exhibited moderate status cribosus, and there were vascular brain injuries with lacunar infarcts observed in the striatum, putamen, and cerebellar white matter. No deposits of TDP43 or alpha-synuclein were detected. Mild to moderate gliosis was observed in the striatum, along with minimal atrophy and neuronal loss. Moderate polyglutamine intranuclear inclusions were observed in caudate and putamen. Amyloid beta staining revealed extensive diffuse plaques, while tau staining demonstrated isolated neurofibrillary tangles and pre-tangles. The observed histological changes in the striatum indicate a Vonsattel stage 2 regarding striatum degeneration. (***Figure 1***)
